# Research Progress on Anti-Inflammatory Effects and Related Mechanisms of Astragalin

**DOI:** 10.3390/ijms25084476

**Published:** 2024-04-19

**Authors:** Jingya Ruan, Zhongwei Shi, Xiaoyan Cao, Zhunan Dang, Qianqian Zhang, Wei Zhang, Lijie Wu, Yi Zhang, Tao Wang

**Affiliations:** 1 State Key Laboratory of Component-Based Chinese Medicine, Tianjin University of Traditional Chinese Medicine, 10 Poyanghu Road, West Area, Tuanbo New Town, Jinghai District, Tianjin 301617, China; ruanjingya@tjutcm.edu.cn (J.R.); cxysssl@163.com (X.C.); dangzhunan998@163.com (Z.D.); qianqian_z0906@163.com (Q.Z.); 2 Tianjin Key Laboratory of TCM Chemistry and Analysis, Tianjin University of Traditional Chinese Medicine, 10 Poyanghu Road, West Area, Tuanbo New Town, Jinghai District, Tianjin 301617, China; 18322306842@163.com (Z.S.); zhangwei940905@163.com (W.Z.); wulj0816@163.com (L.W.)

**Keywords:** astragalin, in vitro anti-inflammatory effects, in vivo anti-inflammatory effects, mechanism of action

## Abstract

Chronic inflammation is a significant contributor to the development of cancer, cardiovascular disease, diabetes, obesity, autoimmune disease, inflammatory bowel disease, and other illnesses. In the academic field, there is a constant demand for effective methods to alleviate inflammation. Astragalin (AST), a type of flavonoid glycoside that is the primary component in several widely used traditional Chinese anti-inflammatory medications in clinical practice, has garnered attention from numerous experts and scholars. This article focuses on the anti-inflammatory effects of AST and conducts research on relevant literature from 2003 to 2023. The findings indicate that AST demonstrates promising anti-inflammatory potential in various models of inflammatory diseases. Specifically, AST is believed to possess inhibitory effects on inflammation-related factors and protein levels in various in vitro cell models, such as macrophages, microglia, and epithelial cells. In vivo studies have shown that AST effectively alleviates neuroinflammation and brain damage while also exhibiting potential for treating moderate diseases such as depression and stroke; it also demonstrates significant anti-inflammatory effects on both large and small intestinal epithelial cells. Animal experiments have further demonstrated that AST exerts therapeutic effects on colitis mice. Molecular biology studies have revealed that AST regulates complex signaling networks, including NF-κB, MAPK, JAK/STAT pathways, etc. In conclusion, this review will provide insights and references for the development of AST as an anti-inflammatory agent as well as for related drug development.

## 1. Introduction

Inflammation is a powerful tool for the innate and adaptive immune systems to maintain cellular and tissue homeostasis [1]. It induces increased vascular permeability, reduced blood rheology, augmented local blood vessel diameter, endothelial cell leakage of white blood cells upon binding to cell adhesion molecules, and the formation of microvascular clots that impede pathogen entry into the bloodstream. Simultaneously, active macrophages release inflammatory mediators that cause chemotactic cells to gather near the lesion (a process known as macrophage aggregation). This sets off a positive feedback loop that intensifies the inflammatory process by releasing additional inflammatory mediators [2]. This defensive reaction is beneficial and necessary under normal circumstances as it can facilitate tissue repair, neutralize sources of infection, and maintain homeostasis, while an excessive response to inflammation may lead to long-term damage and even chronic inflammation. Pro-inflammatory cytokines and pro-inflammatory lipids are vigorously biosynthesized during this process, which propels the ongoing recruitment of immune cells (effector T helper cells, B cells, pro-inflammatory macrophages, etc.). This helps control the inactivation of T regulatory cells in the immune response and ultimately results in aberrant tissue remodeling and irreversible damage [3]. Studies have revealed a close relationship between diabetes, inflammatory bowel disease, cancer, heart disease, arthritis, obesity, and autoimmune disorders with inflammation [4]. It can be seen that how to effectively alleviate inflammation is one of the hottest topics in the academic community.

Anti-inflammatory drugs widely used in clinical practice can be categorized into two types based on their chemical structure. One of them includes steroidal anti-inflammatory drugs such as the glucocorticoid drug dexamethasone, which can exert anti-shock and anti-allergic effects by inhibiting the immune system. However, they have significant side effects, including adrenal cortisol hyperactivity syndrome [5]. Another one is nonsteroidal anti-inflammatory drugs (NSAIDs), such as ibuprofen. Most of these drugs exert antipyretic and analgesic effects by inhibiting prostaglandin (PG) synthesis [6] and are suitable for treating rheumatic diseases, acute or chronic pain, and the prevention of Alzheimer’s disease. However, among many other problems, NSAID side effects also encompass nephrotoxicity, cardiovascular disease, increased blood pressure, and gastrointestinal harm [7].

Studies have shown that natural products contain several effective and low-toxic chemical components, such as flavonoids with strong anti-inflammatory properties found in a variety of fruits, vegetables, and traditional Chinese medicine (TCM) [8]. Flavonoids exist in nature as aglycones, glycosides, and methylated derivatives, each with distinct structures contributing to their anti-inflammatory and free radical scavenging properties. For example, the extraordinary anti-inflammatory properties of quercetin (a flavonoid) are attributed to the presence of the catechol group, whereas the anti-inflammatory properties of morin are inhibited by the insertion of a single hydroxyl group in its B ring [9,10]. These substances exhibit powerful anti-inflammatory properties mediated through a number of pathways, including the inhibition of transcription factors and regulatory enzymes, and have attracted more and more attention. Therefore, searching for flavonoids with minimal side effects and good anti-inflammatory activities from natural products will be one of the hotspots in the research and development of inflammatory inhibitors.

**Figure 1:**
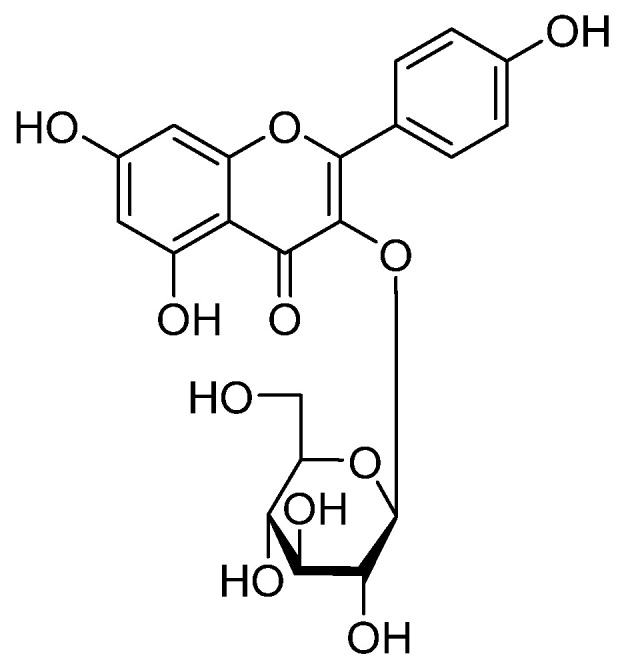
The structure of AST.

Astragalin (AST, Figure 1), also known as kaempferol-3-*O*-glucoside, is a very common natural flavonoid possessing anti-inflammation [11], antioxidant [12], anti-tumor [13], neuroprotection [14], osteoporosis prevention [15], coagulation promotion [16], and other functions. AST is the main component of various TCM commonly used for anti-inflammation, including *Astragalus memeranaceus* (Fisch.) Bge. Var. *mongholicus* (Bge.) Hsiao [17,18], *Morus alba* L. [19,20], *Cuscuta australis* R.Br. [21], *C. chinensis* [22], and *Centella asiatica* (L.) Urb. [23,24]. In Europe, fruits like *Rubus caesius* L. [25] and *M. alba* fruit [26,27] have also been found to contain AST, whose outstanding biological activity and therapeutic effects on various diseases have become a research hotspot in recent years.

Although Riaz et al. [28] and Chen et al. [29] have provided a detailed review of the natural sources, biological activities (antioxidant, neuroprotective, etc.), molecular mechanisms, and pharmacokinetics of AST, there is a lack of systematic reviews on its anti-inflammatory activity and related mechanisms. Therefore, this article focuses on the anti-inflammatory effect of AST, conducts research on relevant literature from 2003 to 2023, and reviews the alleviating effects and molecular mechanisms of AST on various inflammation-related cell and animal models, aiming to provide ideas and references for its subsequent research and the development of related anti-inflammatory drugs.

## 2. Materials and Methods

The review systematically collected references on the pharmacology of Astragalin up to January 2024 from various electronic databases, including Google Scholar, PubMed, and SciFinder. The search terms “Astragalin” OR “Flavonoid astragalin” were combined with “Anti-inflammatory” OR “Anti-inflammation”. Each term was searched using Medical Subject Headings (MeSH) and keyword searches without language limitations. To evaluate relevance, each author independently reviewed English-language publication titles and abstracts. Only the most relevant articles were included after comparing outcomes. Opinions, letters, and comments were excluded from the search.

## 3. The In Vitro Study on the Anti-Inflammatory Effect of Astragalin

The anti-inflammatory potential of AST has been validated in various in vitro models, including macrophages, microglia, and epithelial cells. This article categorizes the cell types employed in these in vitro models, summarizes the impact of AST on inflammation-related factors and protein levels across different cell types, and investigates the role of AST in alleviating inflammation within these in vitro models.

### 3.1. The Effect of Astragalin on Macrophage Inflammation-Related Factors and Protein Levels

Macrophages play a pivotal role in the inflammatory response, undergoing phenotypic and functional changes as the response progresses and being present in various tissues. Activated macrophages can be classified as M1 phenotype macrophages (pro-inflammatory type), M2 phenotype macrophages (anti-inflammatory type), CD169^+^ macrophages, TCR^+^ macrophages, and tumor-associated macrophages. The M1 and M2 phenotypes are more prevalent in inflammatory tissues among them. In order to aid the host in fighting infections, macrophages that have been activated to the M1 phenotype—which can be brought on by lipopolysaccharides—secrete inflammatory and chemotactic proteins when tissues become inflamed. Subsequently, macrophages replace the activated M2 phenotype to repair damaged tissues [30]. There are many types of macrophage models used for the drug’s anti-inflammatory effect study, including mouse macrophages RAW264.7 and J774A.1 cells, human THP1 monocytes, and mouse BMDM bone marrow cells.

It was reported that AST exerted anti-inflammatory effects by antagonizing the increase of nitric oxide (NO), tumor necrosis factor-α (TNF-α), PGE2, interleukin (IL)-1β, IL-6, IL-12, IL-13, IL-18, and leukotriene B4 (LTB4) in the lipopolysaccharide (LPS) and interferon-γ (IFN-γ)-stimulated RAW264.7 cells [31,32,33,34]. In LPS-induced mouse macrophage J774A.1 cells, AST significantly decreased the pro-inflammatory cytokines TNF-α, IL-1β, IL-6, and chemokine macrophage inflammatory protein-1α (MIP-1α), as well as the gene expression of macrophage chemoattractant protein-1 (MCP-1). In addition, AST downregulated the gene expression and protein synthesis of cyclooxygenase (COX) and inducible nitric oxide synthase (iNOS) [35]. Research has shown that the inhibitory effect of AST on NO generation was in a concentration-dependent manner. Its anti-inflammatory potential was similar to that of the iNOS inhibitor L-N6-(1-Iminoethyl)-L-lysine dihydrochloride (L-NIL) [36], suggesting that its anti-inflammatory effect was exerted by inhibiting iNOS.

### 3.2. The Effect of Astragalin on Inflammation-Related Factors and Protein Levels in Microglia

Microglia are distributed throughout the central nervous system and constitute a subset of mononuclear phagocytes comprising 5–10% of all glial cells. As immune effector cells resident in the central nervous system, microglia and their mediated neuroinflammation play a crucial role in the progression of central nervous system injury and disease [37].

Research has shown that when neuroinflammation occurs, iNOS levels increase, inducing NO synthesis from L-arginine in the brain. Subsequently, NO activates COX, leading to an up-regulation of PGE2 levels, and then prostaglandin E synthase 2 (PTGES2) catalyzes the conversion of prostaglandin H2 (PGH2) to PGE2. While it was found that AST inhibited the high expression of iNOS, COX-2, and PTGES2, it reduced TNF-α, IL-1β, and IL-6 levels in LPS-induced microglia, thereby blocking the development of neuroinflammation [38,39]. Additionally, the results demonstrated that AST raised the 1,1-diphenyl-2-picrylhydrazyl radical scavenging as well as the level of heme oxygenase-1 (HO-1) and prevented LPS-induced reactive oxygen species (ROS) formation in microglia, suggesting that AST’s anti-neuroinflammatory actions may be mediated through its prevention of oxidation.

### 3.3. The Effect of Astragalin on Inflammation-Related Factors and Protein Levels in Epithelial Cells

Adenovirus 12-SV40 hybridization and continuous passage immortalization were used to create the normal human bronchial epithelial cell line BEAS-2B, which was obtained from human bronchial epithelial tissue free of illness. It made in vitro studies on the pharmacology, toxicology, and biology of respiratory epithelial cells more convenient [40,41]. In BEAS-2B cells treated with H_2_O_2_, the expressions of intercellular adhesion molecule-1 (ICAM-1), MCP-1, and their integrated proteins were clearly elevated, while AST could dose-dependently decrease their expression [42]. In addition, AST could indirectly weaken the high expression of PLCγ1, PKCβ2, p22phox, and p47phox induced by LPS or H_2_O_2_ by down-regulating the expression of toll-like receptor 4 (TLR4) and eotaxin-1. As a result, the respiratory condition linked to asthma was much improved, and the buildup of intracellular oxides in epithelial cells brought on by LPS was stopped [43].

Furthermore, AST regulates inflammatory factors and proteins associated with cell models, including human gingival epithelial cells, small intestinal epithelial cells (IEC-6), and human immortalized keratinocytes (HaCaT). For example, different concentrations of AST could inhibit the production of TNF-α, IL-6, and IL-1β in IFN-γ-induced HaCaT cells [44], significantly improve the survival rate of LPS-induced IEC-6 cells in rats, and inhibit the mRNA expression level of the inflammatory factors TNF-α and IL-6 [45].

### 3.4. The Effect of Astragalin on Inflammation-Related Factors and Protein Levels in Other Types of Cells

In patients with rheumatoid arthritis (RA), synovial tissue—which is made up of an inner and a lower lining layer—is the primary location of joint inflammation. The inner lining layer is composed of thin fibroblast-like synovial cells (FLS) and macrophages, whereas the lower lining layer is composed of vascularized connective tissue [46]. FLS is the main effector of synovial tissue proliferation and inflammation in RA, with biological characteristics of abnormal proliferation, migration, adhesion, invasion, and secretion, playing an important role in the pathology of RA. Thus, it is possible to effectively regulate the growth of synovitis, stop cartilage degradation, and eventually slow down the evolution of RA by preventing the malfunction of RA synovial cells [46]. Matrix metalloproteinase (MMP) is a regulator of various physiological processes in the body. It is involved in inflammation, angiogenesis, and other processes [47]. According to research, MMP-deficient animals developed severe autoimmune inflammation, indicating that MMPs may have significant immunomodulatory effects [48]. AST was reported to reduce the mRNA and protein expression of MMP-1, MMP-3, and MMP-13 in TNF-α-induced RA-FLS cells [49]. Which indicated that it could alleviate RA by regulating the expression of MMPs.

Furthermore, AST was demonstrated to have anti-inflammatory properties and be able to suppress the generation of histamine in the human peripheral blood eosinophilic leukemia cell line KU812, which was caused by the cross-linking of high-affinity FcεRI (an IgE receptor) and anti-FcεRIα chain antibody CRA-1 [50] (Table 1).

## 4. The In Vivo Alleviating Effect of Astragalin on Inflammatory Disease Models

**Figure 2:**
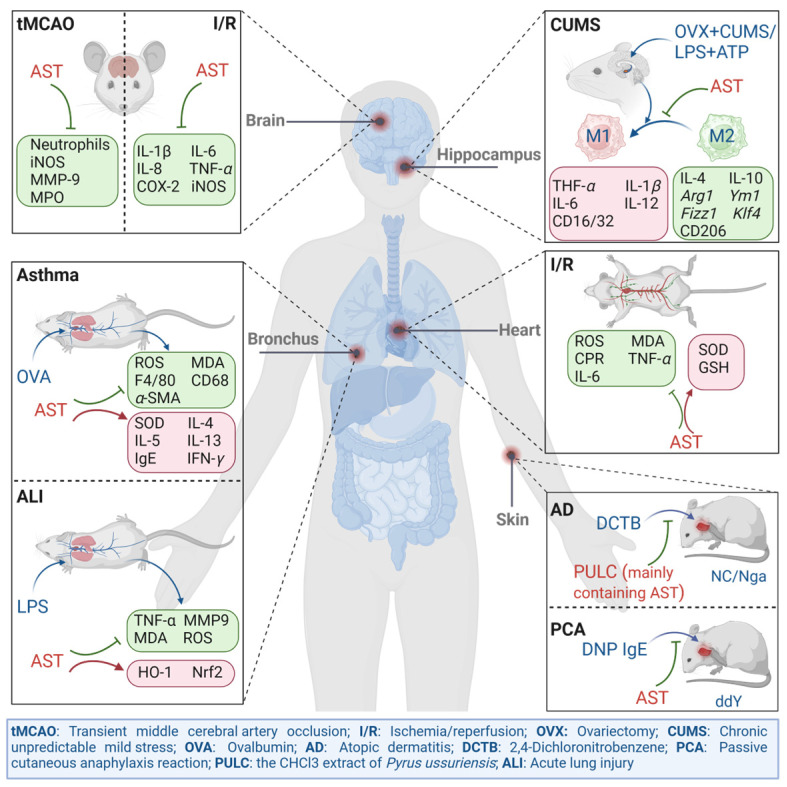
The in vivo alleviating effect of AST on inflammatory disease models (produced by BioRender; license: MC26OIV0X1).

It was reported that AST possessed good therapeutic potential for oxidative stress and inflammation-related diseases, such as atopic dermatitis, allergic respiratory inflammation, ischemia–reperfusion, and depression. These diseases have different pathological characteristics, but their pathogenesis is often accompanied by persistent inflammation. Therefore, this article reviews the in vivo studies of AST on several common disease models, using oxidative inflammation as a clue (Figure 2).

### 4.1. The Effect of Astragalin on Carrageenan-Induced Mouse Foot Swelling Model

Carrageenan is a seaweed polysaccharide used to induce foot swelling in mouse models, which has the advantages of high stability and short duration. It is frequently employed to assess a natural compound’s potential in vivo anti-inflammatory effect [51]. Studies have demonstrated that oral administration of *Annona crassiflora* Mart methanol extract (primarily containing AST) could reduce the amount of total white blood cells in the mice’s pleural cavity caused by modeling and significantly inhibit the formation of hind foot edema induced by carrageenan. It prevented white blood cell migration and plasma leakage after injecting carrageenan into mice’s stomach pockets at a dose of 300 mg/kg [52]. AST has also been proven to alleviate the increase in toe thickness caused by the injection of carrageenan into the soles of mice, and its inhibitory effect is similar to that of the positive drug dexamethasone [34,53]. When physical, chemical, or mechanical damage occurs in the body, the COX pathway is activated, the phospholipid membrane ruptures, and arachidonic acid is produced. Arachidonic acid is further converted into PG analogues through COX, leading to inflammation. After confirming the anti-inflammatory effect of AST on the carrageenan mouse model, Alblihed measured its effect on relevant inflammatory cytokines. The results showed that AST inhibited the activity of COX-2 in inflammatory lesions, downregulated PGE2, IL-1β, IL-6, and nuclear factor κB (NF-κB) levels, significantly reduced the expression of iNOS and the synthesis of NO, and then inhibited inflammatory processes through multiple targets [54].

### 4.2. The Effect of Astragalin on the Atopic Dermatitis Model

Atopic dermatitis (AD) is a chronic inflammatory skin disease that is prone to recurrence and is noninfectious. Its pathological and physiological mechanisms are complex, mainly related to genetics, epidermal barrier defects, abnormal immune responses, and an imbalanced skin microbiota [55]. The CHCl_3_ extract of *Pyrus ussuriensis* Maxim (PULC) is rich in AST, which alleviated specific dermatitis in NC/Nga mice induced by 2,4-dinitrochlorobenzene and significantly reduced scratching behavior and dermatitis score in mice [44]. In order to investigate the anti-allergic effect of AST, researchers established a passive skin allergic reaction (PCA) model. Anti-DNP IgE antibodies were subcutaneously injected into the ear tissue of ddY mice, sensitized for 24 h, and administered by gavage. DNP-BSA antigen was also injected intravenously. After 15 min, the degree of ear swelling was measured. The results showed that AST inhibited dermal thickening, hyperkeratosis, and infiltration of inflammatory cells in PCA mice in a dose-dependent manner and reduced serum IgE levels (without lowering levels of other isotype immunoglobulins such as IgM, IgG1, and IgG2a), exerting an alleviating effect on AD. In addition, AST also inhibited the synthesis of IL-4 and IL-13 in splenic T cells while showing no effect on INF-γ synthesis, indicating that its inhibition of T cell proliferation was not universal [50,56].

### 4.3. The Effect of Astragalin on the Respiratory Inflammation Model

Oxidative stress and pulmonary inflammation are the main pathogenic factors for asthma and chronic obstructive pulmonary disease. Most in vivo studies have used ovalbumin (OVA) to stimulate allergic respiratory inflammation in asthmatic mice. This approach is distinguished by the utilization of distinct allergens, adjuvants, and antigen delivery mechanisms [57]. Based on the above model, Cho et al. found that oral administration of AST could reduce airway collagen fiber deposition and basement membrane fibrosis in OVA-sensitized mice. Further exploration of the inhibitory effect of AST on respiratory cells’s autophagy revealed that AST inhibited the production of ROS and malondialdehyde (MDA) in mouse respiratory tissues induced by OVA, increased the activity of superoxide dismutase (SOD), and thus reduced the damage of oxidative stress to the respiratory tract. Meanwhile, immunohistochemistry experiments confirmed that after OVA modeling, the levels of macrophage markers F4/80 and CD68 in mice increased, while AST treatment significantly reduced their levels to some extent and blocked the infiltration of macrophages and neutrophils. In addition, AST was also reported to reduce the infiltration of mast cells in the respiratory tissue of OVA model mice and lower the level of α-smooth muscle actin (α-SMA) in the lung tissue of OVA model mice, as a result of relieving bronchitis and alleviating ROS-induced bronchial fibrosis [58]. In addition, AST inhibited the increase in eosinophil count and restored the levels of IL-4, IL-5, IL-13, IgE, and IFN-γ in bronchoalveolar lavage fluid (BALF). The above results suggested that AST could inhibit the immune response of Th2 cytokines in an OVA-induced allergic respiratory inflammation mouse model of asthma [59,60].

Acute lung injury (ALI) is usually caused by excessively released oxidants, proinflammatory mediators, and proteolytic enzymes by activated neutrophils during the infection process [61]. Therefore, anti-inflammatory and antioxidant drugs are preferred for treatment. In the LPS-induced ALI rat model conducted by Zheng et al. [12], AST was found to exhibit inhibitory effects on the levels of TNF-α and MMP-9, as well as the markers for oxidative stress, MDA and ROS, while promoting the activity of HO-1. The molecular experiment results showed that the anti-inflammatory and antioxidant activity of AST was closely related to its activation of the Nrf2/HO-1 pathway.

### 4.4. The Effect of Astragalin on the Ischemia–Reperfusion Injury Model

Ischemia reperfusio (I/R) injury refers to secondary damage that occurs to organs and tissues when blood flow is restored following an extended period of ischemia. While the precise etiology of I/R injury remains unclear, existing research indicates that oxidative stress and local inflammatory responses frequently coexist with its damage. The ischemia process induces inflammation already, and the restoration of oxygen and blood flow increases ROS generation and triggers the inflammatory signaling pathway. Damage-associated molecular patterns (DAMP) can be induced by dead cells, particularly necrotic cells, which can mobilize and initiate aseptic inflammatory responses. These reactions mostly appear as an overregulation of pro-inflammatory molecules like TNF-α and IL-6 [62,63].

Studies have demonstrated that AST effectively restored serum levels of glutathione (GSH) and SOD activity, downregulated MDA content, and decreased intracellular ROS levels in rats with spinal cord and cardiac I/R injuries. At the cytokine level, AST markedly lowered C-reactive protein (CPR) and TNF-α in both model rats. The expression of IL-6 was also obviously reduced. Thereby, AST alleviated the inflammatory response caused by oxidative stress [64,65,66]. Furthermore, AST also showed promise in improving brain I/R injury. AST significantly decreased the amount of neutrophil infiltration in brain tissue and the expression levels of the inflammatory markers myeloperoxidase (MPO), MMP-9, and iNOS in a temporary middle cerebral artery occlusion (tMCAO) rat model [67]. RT-qPCR technology was used to detect the expression of the inflammatory cytokines IL-1β, IL-6, IL-8, and TNF-α in a brain I/R injury model rat. The mRNA expression levels of COX-2 and iNOS were significantly downregulated in the treatment group, indicating that AST might be involved in the regulation of some inflammatory genes in the brain I/R injury model [68].

### 4.5. The Effect of Astragalin on the Depression Model

The development of depression and the use of antidepressants are significantly influenced by the reduction of neuroinflammation. Numerous anti-inflammatory medications may have antidepressant benefits, according to clinical research findings, and similar findings have also been supported by animal trials [69]. Microglia can be identified by their distinctive calcium-binding protein, ionized calcium-binding adapter molecule 1 (IBA1), which is only expressed by microglia in the central nervous system. Tong et al. established a chronic unpredictable stress (CUMS) model using female C57BL/6 mice to evaluate the potential regulatory effect of AST on the activation of microglia. Results from immunofluorescence labeling suggested that AST might prevent microglia from activating by reducing the number of IBA1-positive cells [70].

In ovariectomy (OVX) combined with CUMS or LPS combined with ATP rat models, the ELISA test results showed that AST diminished the production of TNF-α, IL-1β, IL-6, and IL-12 in the brain tissue. It was also found that the M1 phenotype (pro-inflammatory) of microglia in the model rat brain was induced to transform into the M2 phenotype (anti-inflammatory), thereby increasing the concentration of anti-inflammatory factors such as IL-4 and IL-10 in the brain. Subsequently, PCR technology was used to detect M2-related genes, and it was found that, compared with the sham surgery group, the mRNA expression of *Arg1*, *Ym1*, *Fizz1*, and *Klf4* was significantly increased after administration of AST, further confirming the promoting effect of AST on the polarization of M2 microglia. The results of the immunofluorescence test demonstrated that, in the hippocampus dentate gyrus of OVX combined with CUMS model rats, AST dramatically decreased the number of co-stained cells of IBA-1, a particular microglia promoter, and CD16/32, an M1 phenotypic microglial marker. The amount of co-stained cells expressing the M2 phenotypic microglial marker, CD206, and IBA1, on the other hand, did not significantly alter, suggesting that AST could both accelerate the polarization of M2 microglia and inhibit that of M1 microglia in order to reduce neuroinflammation [71].

## 5. The Regulatory Effect of Astragalin on Inflammation-Related Pathways

**Figure 3:**
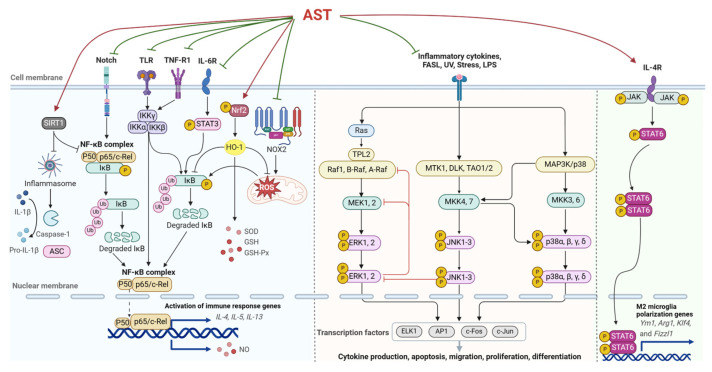
The regulatory effect of AST on inflammation-related pathways (produced by BioRender; license: HI26OIVC9P).

AST has a wide range of pharmacological effects. Through regulating relevant targets like NF-κB, activator protein-1 (AP-1), STAT, nuclear factor E2 related factor 2 (Nrf2), and other transcription factor levels, it can inhibit NF-κB, MAPK, janus kinase/signal transducer, and activator of transcription (JAK/STAT) signaling pathways to alleviate oxidative stress and inflammatory processes (Figure 3). This article reviews the regulatory mechanisms of AST in complex signal networks from different perspectives.

### 5.1. Astragalin Exerts Anti-Inflammatory Effects by Affecting the NF-κB Signaling Pathway

The NF-κB/Rel protein family is a dimeric transcription factor family that includes RelA/p65, RelB, c-Rel, NF-κB2 p52/p100, and NF-κB1 p50/p105. It is essential for controlling the inflammatory response, including innate and adaptive immunity, as well as the survival, proliferation, and differentiation of cells [72]. There are classical and nonclassical NF-κB pathways. A p50-p65-IκB trimer is formed during the active process of the classical signaling pathway when NF-κB, which is coupled with an inhibitory factor of NF-κB (IκB), is in a stationary state in the cytoplasm. The inhibitor of kappa B kinase (IKK) complex, which is made up of IKKα, IKKβ, and NEMO, is activated in response to external stimuli such as growth factors and pro-inflammatory cytokines such as LPS, ILs, body peroxides, and TNF. This causes the active IKKβ to phosphorylate and degrade IκBα protein, which releases NF-κB dimers (p50, p65) from cytoplasmic inhibition and causes them to undergo nuclear translocation. NF-κB-inducing kinase (NIK) stimulates IKKα’s phosphorylation upon activation of non-classical pathways. IKKα then proceeds to phosphorylate the p100 protein, resulting in the phosphorylation of p52 and the release of RelB, producing dimers (p52, RelB) that translocate to the nucleus [73]. Target gene expression is induced by the entry of p50, p52, p65, and RelB into the nucleus, either by themselves or in conjunction with other transcription factors.

Macrophages are the primary source of TNF-α, which binds to TNF-R1 receptors on cell membranes to activate the NF-κB pathway and cause an inflammatory response [74]. Through an electrophoretic mobility shift assay (EMSA), Han et al. found that in the TNF-α-stimulated HCT-116 cell, the NF-κB extract from the nucleus exhibited strong DNA binding activity, while its binding activity was significantly reduced after AST pretreatment [75]. The effect of TNF-α on the nucleus shift of NF-κB was investigated further. It was observed that TNF-α stimulation of HCT-116 cells for two hours led to an increase in cytoplasmic p65 production, followed by nuclear translocation four hours later, indicating a potential mediation of NF-κB p65 nuclear shift by TNF-α. In contrast, AST time-dependently decreased P65 expression in the nucleus in the treatment group [76]. These results indicated that AST might suppress TNF-α-induced transcriptional activity to inhibit the NF-κB pathway.

It was found that AST exhibited anti-inflammatory effects by reducing the elevation of NF-κB protein levels induced by LPS in various cells. For instance, the amount of NO produced in J774A.1 cells stimulated by LPS was decreased by directly blocking the levels of the NF-κB protein [35]. In LPS-induced rat small intestinal epithelial cells, mouse mammary epithelial cells, and mastitis mouse models, AST was also indicated to play a role in inhibiting the phosphorylation of IκBα, IKKα/β, and p65 proteins, thus blocking the nuclear translocation of NF-κB [45,77,78].

LPS and TNF-α have the potential to activate NF-κB via many routes. The upstream regulator cell membrane receptors TLR4 and Notch, STAT3, Nrf2, NADPH oxidase 2 (Nox2), and silent mating type information regulation 2 homolog-1 (SIRT1) will be activated following pro-inflammatory cytokines such as LPS, IL, body peroxides, TNF, and growth factors stimulating. This will alter the levels of downstream genes and proteins.

#### 5.1.1. Astragalin Exerts Anti-Inflammatory Effects by Affecting the TLR4/NF-κB Signaling Pathway

The transmembrane protein TLR4, which is expressed exclusively on the surface of monocytes, is capable of recognizing external stimuli like LPS. By turning on the traditional NF-κB pathway, it can start intracellular signal transduction and release other inflammatory mediators, like NO [79].

Studies have demonstrated that in mice with DSS-induced acute colitis, AST not only markedly decreased the mRNA expression of NF-κB, IKKβ, and IκB but also suppressed the amount of TLR4 mRNA expression in colon tissue. Consequently, AST prevented the onset of inflammation by blocking the TLR4/NF-κB pathway [80]. Hu et al. discovered that AST lowered the protein ratios of *p*-IκBα/IκBα and *p*-NF-κB/NF-κB and downregulated TLR4 protein production in an *Escherichia coli*-induced sheep endometrial epithelial cell culture [81]. Zhao et al.’s study showed that after treating THP-1 macrophages with Parthenolide (an inhibitor of NF-κB), the inhibitory effect of AST on LPS-stimulated inflammatory cytokine release was reversed. Subsequently, they also found that knocking down ATP-binding cassette transporter A1 (ABCA1) and ABCG1 genes using siRNA technology resulted in AST being unable to further inhibit NF-κB nuclear translocation and secretion of inflammatory factors, and the effect of AST on TLR4 expression on the cell membrane surface was eliminated as well [82], which prompted the inhibitory effect of AST on the TLR4/NF-κB pathway depending on its regulation of ABCA1 and ABCG1.

#### 5.1.2. Astragalin Exerts Anti-Inflammatory Effects by Affecting the Notch/HES-1-NF-κB Signaling Pathway

Complex cross-talk mechanisms are also present in the NF-κB and Notch pathways. While earlier research has demonstrated that Notch can raise the mRNA transcription level of all NF-κB family proteins, NF-κB can also raise the mRNA transcription of proteins that are targets of the Notch pathway, including Deltex 1 and Hes [83].

Hu et al. found that AST regulated the Notch/HES-1-NF-κB signaling pathway to weaken the inflammatory response induced by AlCl_3_/D-galactose (Gal). During this process, the expression of Notch1, RBP-Jκ (a DNA binding protein), Hes-1, and NF-κB protein reduced significantly. This substantial reduction in protein levels shielded brain neurons from inflammatory damage by preventing the overactivation of microglia [84].

#### 5.1.3. Astragalin Exerts Anti-Inflammatory Effects by Affecting the STAT3/NF-κB Signaling Pathway

In neuroinflammation, the activation of STAT3 and NF-κB is one of the regulatory mechanisms of IL-6 and TNF-α [85]. Yu et al. demonstrated through immunohistochemical staining experiments that AST could inhibit the nuclear translocation of STAT3 and NF-κB, thereby reducing the phosphorylation levels of STAT3 at Tyr705 and p65 at Ser536, leading to the inhibition of pro-inflammatory mediator expression via regulation of the STAT3/NF-κB pathway [67].

#### 5.1.4. Astragalin Exerts Anti-Inflammatory Effects by Affecting the Nrf2/HO-1/NF-κB Signaling Pathway

One of the significant antioxidants regulated by Nrf2 in the body is HO-1, which is activated by oxygen stress. Additionally, Nrf2 is an antioxidant transcription factor that has anti-inflammatory properties by inhibiting the NF-κB pathway [86,87].

Chen et al. established a rat model of focal brain I/R injury using the suture-occluded method. It was discovered that AST dramatically decreased the number of mothers against decapentaplegic (MAD) in rat brain tissue following an intraperitoneal administration. Its effect on antioxidant stress was linked to encouraging the production of Nrf2 and HO-1 proteins, which raised SOD, GSH, and GSH-Px levels and reduced ROS-induced oxidative tissue damage [88]. In vitro studies have also shown that AST restored the expression of Nrf2 and HO-1 proteins in LPS-induced microglia and inhibited the expression of NF-κB pathway target genes [39]. According to the aforementioned findings, AST could reduce inflammatory damage and strengthen the tissue’s resistance to oxidative stress in the brain I/R model via blocking the Nrf2/HO-1/NF-κB pathway.

#### 5.1.5. Astragalin Exerts Anti-Inflammatory Effects by Affecting the NOX2/ROS/NF-κB Signaling Pathway

Numerous transcription factors can be activated by oxidative stress, which can result in the differential activation of several genes in the inflammatory pathway. The major redox-sensitive transcription factor NF-κB interacts with IκB in the cytoplasm in an inactive form, and reactive oxygen species (ROS) have the ability to cause IκB protein breakdown, which in turn triggers the NF-κB pathway [89]. Nox2, as a part of the NADPH oxidase complex, is the main source of mitochondrial ROS [90]. p47phox is a subunit of Nox2, which is involved in the activation of Nox2 and regulating ROS production [91].

Ma et al. investigated the mechanism of AST’s mitigation of oxidative stress signaling pathways after elucidating its regulatory effects on ROS, MAD, and SOD in model mice. Western blot analysis revealed that AST may lower the expression of Nox2 and p47phox in model mice’s lung tissue, indicating that by blocking the NOX2/ROS pathway, AST may lessen the harm that oxidative stress causes to the body. It was also discovered that AST reduced the expression of IκBα, inhibited the phosphorylation of IκB and NF-κB p65 protein, and suppressed NF-κB nuclear translocation, which resulted in the downregulation of target genes like IL-4, IL-5, and IL-13. These findings suggest that AST may reduce oxidative stress by blocking the NOX2/ROS/NF-κB pathway and thus exerting its anti-inflammatory properties [92].

#### 5.1.6. Astragalin Exerts Anti-Inflammatory Effects by Affecting the SIRT1/NF-κB Signaling Pathway

SIRT1, a NAD^+^-dependent deacetylase, inhibits NF-κB by deacetylating p65 protein subunits through lysine residues changed in the transcription regulatory factors pathway [93].

It was reported that SIRT1 regulated the nuclear translocation of NF-κB, which further induced the activation of IL-1β precursor lysis to produce the pro-inflammatory cytokine IL-1β. This in turn induced the related gene expression of the downstream NOD-like receptor thermal protein domain associated protein 3 (NLRP3) inflammasome (composed of NLRP3, ASC, and caspase-1). After the intervention of AST, SIRT1 was activated, NF-κB signal transduction was interfered with, and the protein expression of NLRP3 and ASC was downregulated. As a result, its anti-inflammatory and neuroprotective effects were exerted [70].

### 5.2. Astragalin Exerts Anti-Inflammatory Effects by Affecting the MAPK Signaling Pathway

The mitogen-activated protein kinase (MAPK) pathway is composed of three conserved kinase patterns, including MAPK, MAPK kinase (MKK), and MAPK kinase (MKKK). The MAPK pathway was considered to be closely related to the oxidation reaction. ROS, such as hydrogen peroxide, has been reported to be one of the main activators of it [94]. When the pathway is activated, the three kinases are sequentially activated to regulate cell proliferation, differentiation, and inflammatory processes [95,96]. Extracellular regulated protein kinases (ERK), c-Jun *N*-terminal kinase (JNK), p38/MAPK, and ERK5 are among the four primary branching pathways from which the MAPK pathway is descended. Among them are p38 and JNK, and both are involved in growth, apoptosis, and inflammation. They can bind to the *N*-terminal regulatory sites of various transcription factors (such as AP-1 and ELK-1) and promote their phosphorylation, thereby regulating the gene expression of inflammatory factors. The Ras/Raf protein, an upstream signal of ERK that is primarily involved in controlling cell proliferation and differentiation, is intimately linked to the emergence and progression of malignancies. One unusual MAPK pathway that has been identified recently is ERK5. After receiving signals such as growth factors, mitogens, and environmental stimuli, it can trigger a cascade reaction of the MEK5-ERK5 signaling pathway, regulate gene expression, and play an important role in tumor metastasis [97,98].

According to studies, AST reduced the protein phosphorylation of JNK and p38 MAPK, which in turn inhibited the JNK/p38 MAPK pathway, by reducing the ratio of *p*-JNK/JNK and *p*-p38 MAPK/p38 MAPK in the cartilage tissue of knee osteoarthritis (KOA) mice [99]. Zhang et al. established a model of uterine inflammation using *Leptospira* and found that AST inhibited the phosphorylation of p38 MAPK, JNK, and ERK in primary endometrial epithelial cells and mouse uterine tissue [100]. Furthermore, in thrombin-induced mouse alveolar tissue, the inhibitory effect of AST on p38 MAPK, JNK, and ERK protein phosphorylation in the MAPK pathway was also confirmed. This effect can be attained by upregulating the levels of PAR-1 and PAR-2 [101].

By binding to VEGF receptors on the membrane of endothelial cells, vascular endothelial growth factor (VEGF) can initiate the conventional MAPK pathway, which in turn activates ERK. The lowering of VEGF levels in tissues by AST may be associated with its inhibition of ERK1/2 phosphorylation in the atherosclerosis model [102]. Kim et al. discovered that by preventing the phosphorylation of LPS-induced upstream protein kinase B (Akt) in microglia, AST can prevent the activation of JNK and p38 [38]. The transcription activating factor AP-1, a heterodimer made up of c-Fos and c-Jun, was continuously phosphorylated by the active JNK, enabling it to reach the nucleus and control target gene expression [103]. While AST had no effect on the phosphorylation of c-Fos during this process, it dramatically reduced the expression and nuclear translocation of the *p*-c-Jun protein, which in turn prevented the production of MMPs and the release of inflammatory cytokines by blocking the JNK/AP-1 pathway [49].

In inflammatory osteolysis, the receptor RANK on osteoclast precursor cells is bound by the osteoclast differentiation factor receptor activator of nuclear factor-κB ligand (RANKL), which activates multiple signaling pathways, including the MAPK pathway. This causes inflammation and increases the formation of downstream molecules that can induce osteoclast fusion and differentiation [104]. Therefore, treating inflammatory osteolysis requires anti-inflammatory treatment first. AST was found to inhibit RANKL-induced phosphorylation of ERK, JNK, and p38 in an inflammatory osteolysis mouse model of the skull. It was also found to block the downward transmission of MAPK pathway signals, which in turn suppressed the levels of downstream proteins such as c-Fos, nuclear factor of activated T cells cytolytic 1 (c1NFATC1), cathepsin K (CTSK), and tartrate-resistant acid phosphatase (TRAP). This led to a remission of inflammatory osteolysis [105].

### 5.3. Astragalin Exerts Anti-Inflammatory Effects by Affecting the IL-4R/JAK1/STAT6 Signaling Pathway

After binding to IL-4 and IL-4R, the human tyrosine kinase JAK1 forms and phosphorylates a complex with an active receptor. It then phosphorylates STAT6 once again in order to activate it. As a result, it changes its ex vivo conformation, forms a polymer, and travels to the nucleus, where it regulates the transcription of genes associated with M2 microglia polarization [106]. Research has demonstrated that there was a suppression of the IL-4R/JAK1/STAT6 pathway and low levels of anti-inflammatory factors in the tissues affected by neuroinflammation. AST demonstrated suppressive effects on neuroinflammation by upregulating the expression of *p*-JAK1 and IL-4R, enhancing the intracellular level of *p*-STAT6, and promoting the polarization of M2 microglia. These actions also prevented the degradation of STAT6 by proteasomes through ubiquitination [71].

## 6. Discussion

As we all know, long-term inflammation will cause many chronic diseases, such as heart disease, diabetes, etc. AST, as one of the flavonoids widely present in natural plants, showed various activities, including anti-inflammatory, antioxidant, and neuroprotective effects in animals. Therefore, it is not only a drug with the potential to treat inflammatory diseases but also a safe dietary supplement. AST has relatively low cytotoxicity and side effects and can alleviate inflammation progression in vivo through multiple targets and pathways, which gives it certain advantages in the treatment of some chronic inflammatory diseases.

The mechanism of AST’s anti-inflammatory activity varied a lot, according to the above summary. While the NF-κB pathway is a typical abnormal activation signaling pathway in inflammation-related diseases, it is common to different inflammatory-based diseases such as neuroinflammation, I/R injury, and airway inflammation. Moreover, the anti-oxidant effect is supposed to be another common mechanism in AST for treating atopic dermatitis, allergic respiratory inflammation, ischemia–reperfusion, and depression.

However, due to the poor water solubility and low bioavailability of AST, it is necessary to enhance the physiological activity of AST through specific chemical modifications. Metabolic engineering technologies such as enzymatic synthesis have been widely utilized for structural modification, resulting in hydrophilic AST galactoside or glucoside with improved water solubility and oxidative stability, as well as enhanced efficacy in scavenging ABTS·^+^ radicals and inhibiting inflammatory cytokines [107,108,109].

We still need to further explore more suitable dosage regimens and adjuvants for AST in the treatment of specific diseases, which may amplify its biological activity in vivo. Additionally, the specific pharmacological mechanisms and clinical safety of AST also need to be further evaluated.

## 7. Conclusions

Inflammation plays an essential role in long-term systemic harm. In summary, AST was considered to show good anti-inflammatory potential in various inflammatory disease models. In brain tissue, AST effectively alleviated neuroinflammation and brain damage and possessed the potential to treat moderate diseases such as depression and stroke. In intestinal tissue, AST exhibited significant anti-inflammatory effects on both large and small intestinal epithelial cells, and animal experiments have also shown that AST exerted good therapeutic effects on colitis mice. Therefore, it is also considered one of the potential drugs for treating ulcerative colitis. AST was also found to alleviate respiratory diseases such as allergic asthma and pneumonia. In addition, AST performed a certain therapeutic effect on mastitis, arthritis, thyroiditis, atherosclerosis, and other diseases, which suggested that AST showed a wide range of anti-inflammatory effects. However, so far, there have been few reports on clinical research on AST, which has greatly limited its application and development, and further related research is urgently needed.

## Figures and Tables

**Table 1:** In vitro study on the anti-inflammatory effect of AST.

Cell Type	Cell	Induction Method	Testing Index	Refs.
Macrophages	RAW264.7	LPS + IFN-γ	NO ↓, TNF-*α* ↓, PGE2 ↓, IL-1*β* ↓, IL-6 ↓, IL-12 ↓, IL-13 ↓, IL-18 ↓, LTB4 ↓; IL-10 ↑	[31,32,33,34]
	J774A.1	LPS	iNOS ↓, COX ↓, TNF-α ↓, IL-1β ↓, IL-6 ↓, MIP-1α ↓, MCP-1 ↓	[35]
Microglia cells	Mice microglia cell	LPS	iNOS ↓, NO ↓	[38,39]
	BV2 microglial cell	LPS	iNOS ↓, COX-2 ↓, PTGES2 ↓, TNF-α ↓, IL-1β ↓, IL-6 ↓, NO ↓	[38]
Epithelial cells	BEAS-2B cell	H_2_O_2_	ICAM-1 ↓, MCP-1 ↓, MCP-1/ICAM-1/αv ↓	[42]
	BEAS-2B cell	LPS/H_2_O_2_	TLR4 ↓, Eotaxin-1 ↓, PLCγ1 ↓, PKCβ2 ↓, p22phox ↓, p47phox	[43]
	HaCaT	TNF-α/IFN-γ	IL-6 ↓, IL-1β ↓	[44]
	IEC-6	LPS	TNF-α ↓, IL-6 ↓	[45]
Other types of cells	RA-FLS cell	TNF-α	MMP-1 ↓, MMP-3 ↓, MMP-13 ↓	[49]
	KU812	FcεRI + CRA-1	Histamine ↓	[50]

Ref.: reference; ↑: up-regulated; ↓: down-regulated.

## Abbreviation

ABCA1: ATP binding cassette transporter A1; AD: atopic dermatitis; Akt: protein kinase B; ALI: acute lung injury; AP-1: activator protein-1; AST: Astragalin; BALF: bronchoalveolar lavage fluid; c1NFATC1: T cells cytolytic 1; COX: cyclooxygenase; CPR: C-reactive protein; CTSK: cathepsin K; CUMS: chronic unpredictable stress; DAMP: damage-associated molecular patterns; EMSA: electrophoretic mobility shift assay; ERK: extracellular regulated protein kinases; FLS: fibroblast-like synovial cells; Gal: galactose; GSH: glutathione; HO-1: heme oxygenase-1; IBA1: ionized calcium-binding adapter molecule 1; ICAM-1: intercellular adhesion molecule-1; IκB: inhibitory factor inhibitor of NF-κB; IKK: inhibitor of kappa B kinase; iNOS: induced nitric oxide synthase; I/R: ischemia reperfusion; JAK: janus kinase; JNK: c-Jun *N*-terminal kinase; KOA: knee osteoarthritis; MAD: mothers against decapentaplegic; MAPK: mitogen activated protein kinase; MCP-1: macrophage chemoattractant protein-1; MDA: malondialdehyde; MIP-1α: macrophage inflammatory protein-1α; MKK: MAPK kinase; MKKK: MAPK kinase; MMP: Matrix metalloproteinase; MPO: myeloperoxidase; NLRP3: NOD-like receptor thermal protein domain associated protein 3; NO: nitric oxide; Nox2: NADPH oxidase2; Nrf2: nuclear factor E2-related factor; IFN-γ: interferon-γ; IL: interleukin-1β; L-NIL: L-N6-(1-Iminoethyl)-L-lysine dihydrochloride; LPS: lipopolysaccharide; LTB4: leukotriene B4; NF-κB: nuclear factors κB; NIK: NF-κB-inducing kinase; NSAIDs: nonsteroidal anti-inflammatory drugs; OVA: ovalbumin; OVX: ovariectomy; PCA: passive skin allergic reaction; PG: prostaglandin; PTGES2: prostaglandin E synthase 2; PULC: The CHCl_3_ extract of *Pyrus ussuriensis* Maxim; RANKL: receptor activator of nuclear factor-κB ligand; ROS: Reactive oxygen species; SIRT1: silent mating type information regulation 2 homolog-1; α-SMA: α-smooth muscle actin; SOD: superoxide dismutase; STAT: signal transducer and activator of transcription; TCM: traditional Chinese medicine; TLR4: toll-like receptor 4; tMCAO: transient middle cerebral artery occlusion; TNF-α: tumor necrosis factor-α; TRAP: tartrate resistant acid phosphatase; VEGF: vascular endothelial growth factor.
